# Identification of *Plasmodium* spp. in Neotropical primates of Maranhense Amazon in Northeast Brazil

**DOI:** 10.1371/journal.pone.0182905

**Published:** 2017-08-10

**Authors:** Mayra Araguaia Pereira Figueiredo, Silvia Maria Di Santi, Wilson Gómez Manrique, Marcos Rogério André, Rosangela Zacarias Machado

**Affiliations:** 1 Laboratory of Immunoparasitology, School of Agrarian and Veterinary Sciences (FCAV), UNESP, Jaboticabal Campus, Jaboticabal, São Paulo, Brazil; 2 Center for Malaria Studies, Superintendency for Endemic Disease Control (SUCEN), Department of Health of the State of São Paulo/ Institute of Tropical Medicine of Sao Paulo (IMTSP), USP, Sao Paulo, São Paulo, Brazil; 3 Laboratory of Veterinary Pathology, Brazil University, Descalvado Campus, Descalvado, São Paulo, Brazil; Centro de Pesquisas Rene Rachou, BRAZIL

## Abstract

In the Brazilian Amazon region, malaria caused by *Plasmodium malariae* is considered to be a zoonosis because of cross-transfer of the parasite between humans and Neotropical primates. To contribute information on this issue, we investigated occurrences of natural infection with *Plasmodium* sp. among Neotropical primates in the Maranhense Amazon (Amazon region of the state of Maranhão), in the northeastern region of Brazil. Blood samples were collected from 161 Neotropical primates of six species that were caught in an environmental reserve (Sítio Aguahy) and from captive primates (CETAS—Wildlife Screening Center, municipality of São Luís), in Maranhão. *Plasmodium* sp. was diagnosed based on light microscopy, PCR, qPCR and LAMP for amplification of the 18S rRNA gene. Serum samples were also assayed by means of indirect immunofluorescence for IgG antibodies against *P*. *malariae*/*P*. *brasilianum*, *P*. *falciparum* and *P*. *berghei*. Parasites were detected through light microscopy on five slides from captive primates (four *Sapajus* spp. and one *Callithrix jacchus*). In the molecular tests, 34.16% (55/161) and 29.81% (48/161) of the animals sampled were positive in the qPCR and PCR assays, respectively. In the PCR, 47/48 animals were positive for *P*. *malariae*/*P*. *brasilianum*; of these, eight were free-living primates and 39 from CETAS, São Luís. One sample showed a band in the genus-specific reaction, but not in the second PCR reaction. Anti-*P*. *malariae*/*P*. *brasilianum* IgG antibodies were detected in four serum samples from *Sapajus* spp. in captivity. In this study, circulation of *P*. *malariae*/*P*. *brasilianum* in Neotropical primates was confirmed, with low levels of parasitemia and low levels of antibodies. The importance of these animals as reservoirs of human malaria in the region studied is still unknown. This scenario has an impact on control and elimination of malaria in this region.

## Introduction

Malaria in humans is the most noteworthy parasitic disease around the world [1]. In 2015, an estimated 212 million cases of malaria occurred worldwide, with 429,000 deaths [2].

Eradication of malaria is therefore a priority for international organizations involved in disease control. Thus, knowledge about simian malaria is essential, given that its presence can put at risk the eradication of human malaria [3,4]. The coevolutionary history of primate malarias and their hosts is matter for concern because of the potential pathogenicity and life-history traits of human malaria, as well as the zoonotic potential of simian malaria. The two most important species of human malaria, *Plasmodium vivax* and *P*. *falciparum*, underwent a cross-species transfer event from non-human primates to humans [5,6], but how long ago this occurred remains unknown. Phylogenetic analyses supports *P*. *vivax* among the Asian primate malarias, being possible that *P*. *vivax* is a zoonosis from Asian monkeys [7]. The human *P*. *falciparum* is of gorilla origin and all known human strains may have resulted from a single cross-species transmission event [6]. More recently, it was reported human malaria cases due to *P*. *knowlesi*, a species that originally infected primates of the genus *Macaca* in Southeast Asia. Currently responsible for the highest percentual among species count of human malaria causing in Malaysia (68%) [8,9].

Two species of *Plasmodium* of Neotropical primates (NTPs), *P*. *brasilianum* and *P*. *simium*, have been found in the Americas. The first species causes quartan malaria, which is widely distributed in the Central America and Amazon region of South America. [10–12], and was first identified in *Cacajao calvus* imported from the Brazilian Amazon region to Germany [13]. *P*. *brasilianum* has been described infecting several taxa of primates (the Atelidae and Cebidae families) and was recently described in specimens of *Saguinus midas niger*, *Saguinus martinsi*, *Leontopithecus rosalia*, *Leontopithecus chrysomelas*, *Callithrix geoffroyi* and *Mico humeralifer*, in the subfamily Callitrichinae [14], according to the classification of Perelman et al. [15]. Specimens of this subfamily are less frequently diagnosed with *Plasmodium* than those of the subfamily Cebinae [14].

*P*. *simium*, in turn, causes tertian malaria and is restricted to NTP species living in the Atlantic Forest in the southeastern and southern regions of Brazil, where it has been reported in the states of Espírito Santo, São Paulo, Santa Catarina and Rio Grande do Sul [16]. Fonseca [17] described it in *Alouatta fusca* in the state of São Paulo. Until now, it has been only identified in the species *Alouatta fusca*, *Brachyteles arachnoides* [18], *Alouatta caraya* [19], *Sapajus xanthosternos*, *Sapajus robustus* and *Cebus* sp. [20].

*P*. *brasilianum* and *P*. *simium* are of particular interest because they are morphologically difficult to distinguish from the human malaria species, *P*. *malariae* and *P*. *vivax*, respectively, and can be transmitted naturally [21,22] or experimentally [14] to humans. In the Amazon region, quartan malaria it has been speculated as a zoonosis, due to the cross-transference of the parasite from rural, indigenous populations and NTP, confirmed by high levels of seropositivity for the antigens of *P*. *malariae/P*.*brasilianum* in both humans and NTP. This would comprise cross-transference of the parasite from NTP populations to rural human populations, and it has been confirmed by high levels of seropositivity for *P*. *malariae/P*. *brasilianum* antigens in humans and NTPs [23,24]. Additionally, similarity of the MSP-1, 18S rRNA and CSP sequences of these two parasite species isolated from human and NTP hosts has been observed [22,25].

NTPs positive for *P*. *brasilianum* were found in three municipalities in the Amazon region of the state of Maranhão in the 1970s. Out of 29 blood samples of primates examined via light microscopy, seven (24.13%) were positive for *P*. *brasilianum* [26]. Recently, new studies using molecular techniques carried out on the island of São Luís (Maranhense Amazon) showed that captive and free-living NTPs were naturally infected with *Plasmodium*, with a positivity rate of 18.57% (13/70) [27]. Regarding human cases of malaria in the state of Maranhão, the number of cases has been decreasing year by year, with a reduction of 61% between 2014 and 2015 [28]. A low number of *P*. *malariae* cases has been notified, with six records from 2014 to June 2016. None of these cases occurred in the municipalities of the island of São Luís (São Luís, São José de Ribamar, Paço do Lumiar and Raposa) [29].

The present study resulted from ongoing research on simian malaria in the state of Maranhão. Its aim was to identify *Plasmodium* in blood samples from free-living and captive NTPs in two municipalities on the island of São Luís, in the state of Maranhão, northeastern Brazil, obtained between 2009 and 2014.

## Materials and methods

### Study areas and blood samples from NTPs

**Fig 1:**
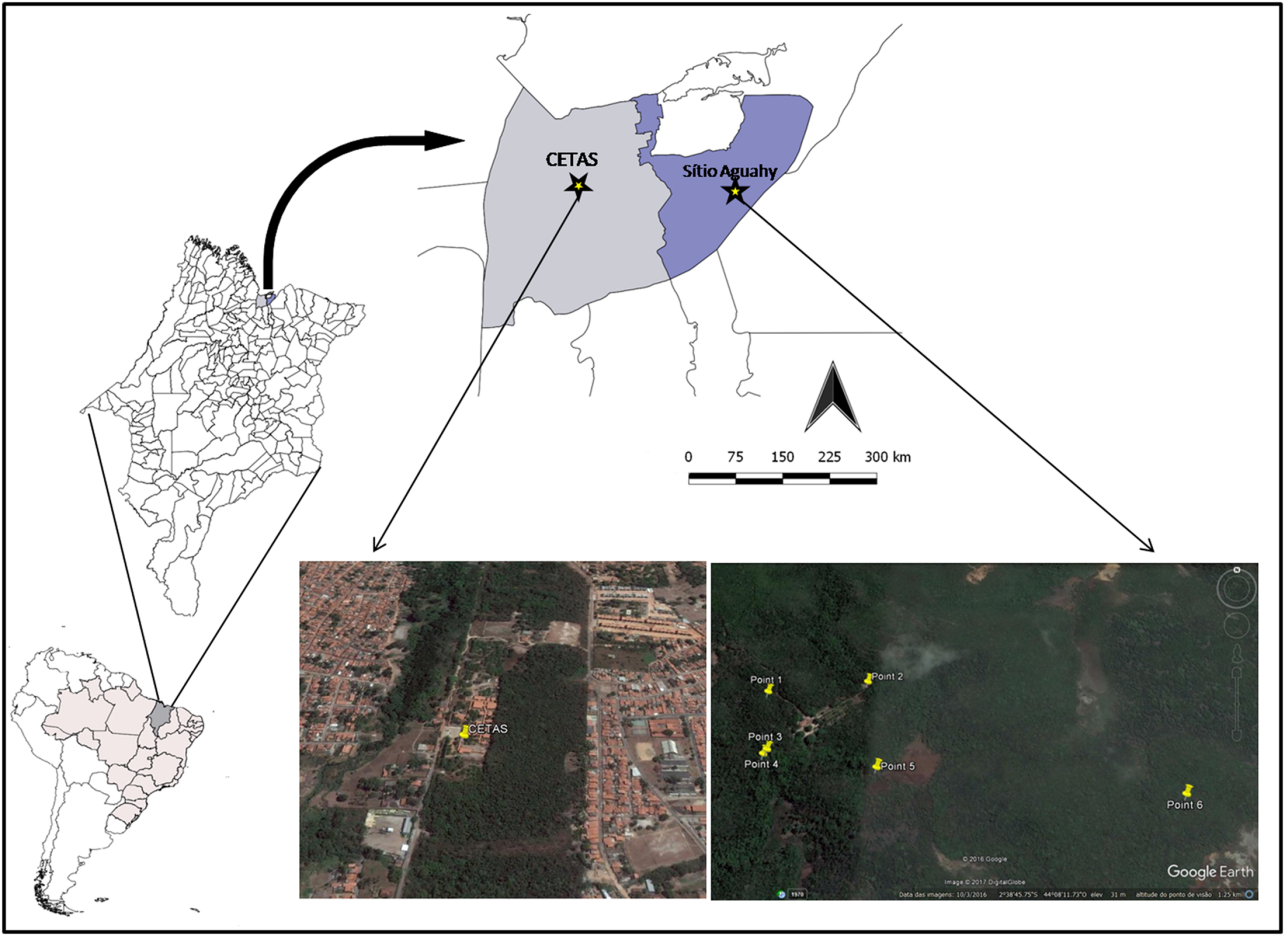
Map of South America highlighting Brazil and the state of Maranhão. Map of the island of São Luís showing the locations of CETAS, in the municipality of São Luís (light blue) and the Sítio Aguahy, municipality of São José de Ribamar (dark blue). Satellite image of CETAS and Sítio Aguahy, which were the capture and collection sites for blood samples from Neotropical primates.

Blood sampling from NTPs was carried out at the Wildlife Screening Center (CETAS, according to its initials in Portuguese), which is located in one of the most populous districts of the municipality of São Luís (Fig 1), and on the Sítio Aguahy, a private preservation area located in the rural zone of the municipality of São José de Ribamar. These two municipalities are part of the island of São Luís, in the north of the state of Maranhão, northeastern Brazil (Fig 1), which lies within the Amazon biome. Blood samples (n = 141) were collected from NTPs at CETAS (2°56'80" S, 44°21'01" W), between 2009 and 2014. A further 20 samples from NTPs were collected in the Sítio Aguahy (2°38'76" S, 44°08'22" W) between 2011 and 2014.

This study was approved by the Ethics Committee for Animal Use of FCAV-UNESP, Jaboticabal Campus (protocol no. 011.480/12) and by the Instituto Chico Mendes de Conservação da Biodiversidade (Chico Mendes Institute for Biodiversity Conservation) (ICMBio) (license no. 34.282–2).

The NTPs (Table 1) were firstly anaesthetized using a combination of tiletamine hydrochloride and zolazepam hydrochloride (Zoletil^®^, Virbac), with proportionate doses for each species. Blood samples from the NTPs were collected by means of venous puncture of the femoral or jugular veins and were transferred to Vacutainer^®^ tubes containing EDTA for molecular tests, and to tubes without anticoagulant for serological tests. In total 151 serum samples and 161 whole blood samples were collected.

**Table 1:** Species of Neotropical primates sampled from 2009 to 2014 in two municipalities on the island of São Luís (São Luís and São José de Ribamar), state of Maranhão, Brazil, to investigate the presence of *Plasmodium*, detailing the scientific name, common name, place of capture and sex.

Species	Common name	Place of capture*	Sex	Total
*Aotus infulatus*	night monkey	CETAS	1F	1
*Chiropotes satanas*	black bearded saki	CETAS	1M	1
*Sapajus* spp.	capuchin	CETAS (89) and Sítio Aguahy (17)	61 M/45F	106
*Callithrix jacchus*	common marmoset	CETAS	12M/7F	19
*Saimiri sciureus*	squirrel monkey	CETAS (21) and Sítio Aguahy (3)	17M/7F	24
*Saguinus midas niger*	black-handed tamarins	CETAS	5M/5F	10
Total			96M/65F	161

*CETAS is located in the municipality of São Luís; Sítio Aguahy is located in the municipality of São José de Ribamar.

The number in parentheses indicates the numbers of primates sampled at each capture site. M-male, F-female.

### Thick blood smear and thin blood smear diagnoses

Thick and thin blood smears were prepared from each specimen, in accordance with WHO recommendations, and were analyzed by means of light microscopy [30]. The blood smears were fixed with methanol and stained with Giemsa (Sigma-Aldrich, St. Louis, USA). The thick blood smears were pre-stained with buffered methylene blue solution and stained with Giemsa (Sigma-Aldrich, St. Louis, USA) [27].

### DNA extraction

DNA was extracted from 200 μL of whole blood from NTPs using the QIAamp DNA mini kit (Qiagen^®^, Valencia, CA, USA), in accordance with the manufacturer's recommendations, with a final elution in 50 μL. The DNA concentration and absorbance ratio (260/280) nm were measured using spectrophotometer equipment (Nanodrop^®^, Thermo Scientific, Waltham, MA, USA).

### qPCR assays and preparation of standard plasmids

The qPCR assays to identify *Plasmodium* based on the 18S rRNA gene [31] were carried out in duplicate, using 25 μL of a mixture containing 12.5 μL of 1X TaqMan Universal Master Mix^®^ (Life Technologies), 0.5 μM of the primers M60 (5’-ACATGGCTATGACGGGTAACG-3’) and M61 (5’-TGCCTTCCTTAGATGTG GTAGCTA-3’), 0.3 μM of the hydrolysis probe M62 (FAM5’-TCAGGCTCCCTCTCC GGAATCGA-3’TAMRA) and 5 μL of DNA [32]. The amplification reactions were carried out on Low-Profile Multiplate^™^ Unskirted PCR plates (BioRad, Hercules, CA, USA) in a CFX96 thermal cycler (BioRad, Hercules, CA, USA). The amplification conditions were 50°C for 2 minutes, 95°C for 10 minutes and 40 cycles of 94°C for 30 seconds and 60°C for 1 minute. The qPCR assays were carried out in accordance with the recommendations of the MIQE Guidelines (Minimum Information for Publication of Quantitative Real-Time PCR Experiments) [33].

DNA control samples for *P*. *falciparum* and *P*. *malariae* [34,35] were amplified by means of conventional PCR, in accordance with the protocol of Gama et al. [31]. The products were purified using a Silica bead DNA gel extraction kit (Thermo Scientific) and were quantified in a spectrophotometer (Nanodrop, Thermo Scientific, Dubuque, Illinois, USA) for in-house preparation of plasmids. Cloning of the purified products was carried out in the pGEM-T Easy Vector system (Promega, Madison, Wisconsin, USA), and the reactions were carried out as recommended by the manufacturer. The products from the ligations were transformed using *Escherichia coli* One Shot Match 1^™^ chemically competent cells (Invitrogen, Carlsbad, California, USA). The resulting clones were subjected to blue/white colony screening. The plasmid DNA was extracted using the QIAprep miniprep kit (Qiagen, Valencia, CA, USA). The number of plasmid copies was determined using the following formula: (Xg/μL of DNA/[plasmid length in bp × 660]) × 6.022 × 10^23^ × copies of plasmid/μL. The amplification efficiency (E) of each assay was calculated from the standard curve using the formula E = 10^−1^/slope. PCR amplifications were conducted on Low-Profile Multiplate^™^ Unskirted PCR Plates (BioRad, Hercules, CA, USA) using a CFX96 thermal cycler (BioRad).

### PCR assay

The semi-nested multiplex PCR protocol for *Plasmodium* was carried out based on the 18S rRNA gene, previously described by Rubio et al. [36]. The primers PLF (5’-AGTGTGTATCAATCGAGTTT-3’), UNR (5’-GACGGTATCTGATCGTCTT-3’) and HUF (5’-GAGCCGCCTGGATACCG-3’) were used. These generated two fragments: one of 231 bp from the mammals’ endogenous gene, which is a small subunit of the ribosomal gene (ssrDNA) generated by the primer pair UNR-HUF; and another of 783 to 821 bp from the 18S rRNA gene of *Plasmodium* (generated by the UNR-PLF primer pair). The reaction was performed in a final volume of 25 μL, containing 10X PCR buffer, 2.5 mM of MgCl_2_, 0.1 mM of a mixture of deoxynucleotide triphosphates, 0.5 μM of PLF, 0.5 μM of UNR, 0.04 μM of HUF, 1.25 U of Taq platinum DNA polymerase (Life Technologies^®^, Carlsbad, CA, USA) and 5 μL of DNA. In all the reactions, ultra-pure water (Promega) was used as a negative control. DNA of *P*. *vivax* and *P*. *malariae* from infected patients [33, 34] and DNA of *P*. *falciparum* (strains K1 and Palo Alto), cultivated *in vitro*, were used as positive controls. The PCR amplifications were carried out at 94°C for 5 minutes, followed by 40 cycles of 94°C for 45 seconds, 62°C for 45 seconds and 72°C for 60 seconds, and a final extension of 72°C for 10 minutes. The products were then subjected to a species-specific reaction using 2 μL of product, amplified for reaction, with a final volume of 25 μL: 1X Taq buffer, 2.5 mM of MgCl_2_, 0.1 mM of deoxynucleotide triphosphate mixture, 0.5 μM of PLF, 0.064 μM of MAR (5’-GCCCTCCAATTGCCTTCT-3’) (269 bp for *P*. *malariae*), 0.3 μM of FAR (5’-AGTTCCCCTAGAATAGTTACA-3’) (395 bp for *P*. *falciparum*), 0.05 μM of VIR (5’-AGGACTTCCAAGCCGAAG-3’) (499 bp for *P*. *vivax*), 1.25 U of Taq DNA polymerase. The amplifications were performed under the following conditions: 94°C for 5 minutes and 35 cycles of 94°C for 20 seconds, 62°C for 20 seconds and 72°C for 30 seconds, and a final extension of 72°C for 10 minutes.

For sequencing of the genus-specific protocol, based on the 18S rRNA gene, the primers and the thermal sequences described by Santos et al. [37] were used. The 240 bp products were viewed via electrophoresis on 2% agarose gel under UV light and were purified using a Silica bead DNA gel extraction kit (Thermo Scientific).

### LAMP (loop-mediated isothermal amplification) assay

The LAMP assay was carried out using the set of genus-specific primers previously described by Han et al. [38]. The reactions were performed using the Loopamp^®^ DNA kit (Eiken Chemical Co. Ltd., Japan), in accordance with Han et al. [38], on 13 blood samples from NTPs that had previously tested positive for *Plasmodium* in the qPCR and PCR assays. The final volume reaction of 25 μL contained 1.6 μM of the FIB and BIP primers, 0.2 μM of the F3 and B3 primers, 0.8 μM of the LFP and LPB primers, 1 μL of Syto 9 (125 μM) (Life Technologies^®^), 2X reaction buffer (12.5 μL), 1 μL of *Bst* DNA polymerase and 2 μL of DNA template.

The reactions were performed at 60°C for 100 minutes, followed by inactivation of the enzyme at 80°C for 2 minutes in a CFX96 thermal cycler (BioRad, Hercules, CA, USA). The dissociation curve was performed at 60–96°C with an increase of 0.5°C every 0.5 seconds.

### Indirect immunofluorescence assay (IFA)

IFA slides were prepared from crude human erythrocyte antigens infected with *P*. *malariae* [34] and *P*. *falciparum* (K1 and Palo Alto strains) from *in vitro* cultures and red cells from BALB/c mice infected with *P*. *berghei* ANKA. The initial dilution for detection of IgG antibodies against the three species was 1:20. All the NTP serum samples were tested for anti-*P*. *falciparum* and anti-*P*. *berghei* IgG antibodies. However, due to difficulty in obtaining blood samples from human patients infected with *P*. *malariae*, only ten NTP serum samples that were positive in the semi-nested multiplex PCR [36] were tested for presence of anti-*P*. *malariae* antibodies. Human serum samples were included as negative and positive controls in all the assays [32,34]. Briefly, 10 μL of the test serums and diluted control serums (1:20) were deposited in PBS at pH 7.2 (130 mM of NaCl, 2.7 mM of KCl, 5.6 mM of Na_2_HPO_4_, 1 mM of KH_2_PO_4_ and 0.8 mM of NaH_2_PO_4_) in each well and were incubated at 37°C for 30 minutes in a humidity chamber. The slides were washed three times in PBS (pH 7.2) by means of immersion (five minutes each). They were dried at room temperature and 10 μL of anti-monkey IgG FITC-conjugation (Sigma-Aldrich^®^, St. Louis, Missouri, USA), diluted in accordance with the manufacturer's instructions, were added to the wells with the test serums, while 10 μL of anti-human IgG FITC (Sigma-Aldrich^®^, St. Louis, Missouri, USA) were added to the wells with the controls. Thereafter, the slides were incubated, washed and dried as described above. The slides were mounted with buffered glycerin (9 mL of glycerin to 1 mL of carbonate-bicarbonate solution, at pH 9.6) and were evaluated under an ultraviolet light-emission microscope (Olympus, BX-FLA).

## Results

### Diagnosis from thick blood smears and thin blood smears

**Fig 2:**
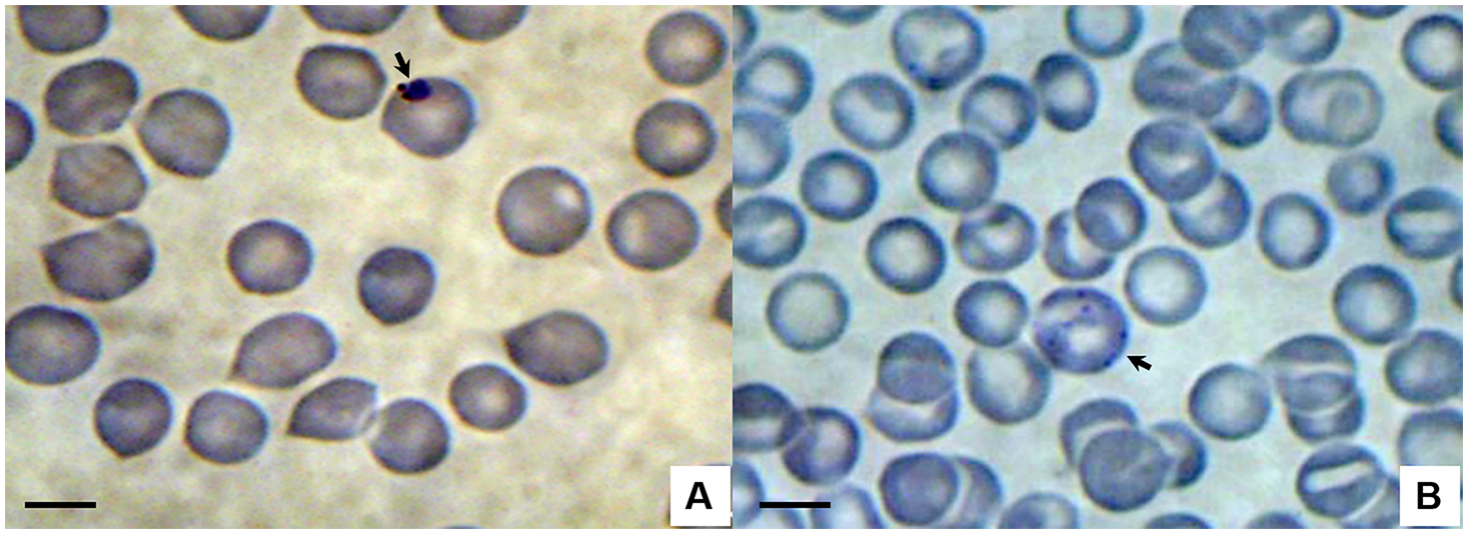
Photomicrographs of *Plasmodium* sp. viewed via light microscopy on thin blood smears from Neotropical primates sampled at CETAS, in São Luís. A—ring-shaped trophozoite in sample from *Sapajus* sp. (male); B—schizont in sample from *Callithrix jacchus* (male). Giemsa staining. Bar = 10 μm.

In total, 322 slides were prepared: 161 thick blood smears and 161 thin blood smears. In the slides from five NTPs (four individuals of *Sapajus* spp. and one of *Callithrix jacchus*) that were being kept at CETAS, in São Luís, structures suggestive of *Plasmodium* were observed (Fig 2).

### Molecular diagnosis

In genus-specific qPCR for *Plasmodium* based on the 18S rRNA gene, 34.16% (55/161) of the animals were positive: seven of them (12.72%) were free-living primates that were caught at the Sítio Aguahy, in São José de Ribamar; and 48 (87.28%) were from CETAS, in São Luís. In the PCR, however, 29.81% (48/161) of the samples were amplified for *Plasmodium*: nine of them (18.75%) were from free-living primates that were caught at the Sítio Aguahy, in São José de Ribamar; and 39 (81.25%) were from CETAS, in São Luís. All the samples that were positive through PCR were also found to be positive through qPCR. However, seven samples from *Sapajus* sp. were positive only through qPCR.

In the species-specific diagnosis through PCR, 47/48 of the animals that had previously been found to be positive in the genus PCR were positive for *P*. *malariae*/*P*. *brasilianum*; of these, eight were free-living primates. One sample showed a band in the genus-specific reaction, but not in the second PCR reaction. Unfortunately, almost all the positive samples produced weak-intensity bands and sequencing was not possible. However, four DNA samples from the NTPs were sequenced. The nucleotide sequences generated from each sample were smaller than 200 bp, which meant that they could not be deposited in GenBank, but they were nonetheless compared with sequences that have been deposited in that database, using BLASTn for similarity analysis [39]. Sample 136 (*Sapajus* sp. from CETAS) was found to be 100% identical to a sequence of *P*. *malariae* (GU950655); sample 137 (*Sapajus* sp. from CETAS) was found to be 97% identical to a sequence of *Plasmodium* ZOOBH (EF090276); sample 145 (*Sapajus* sp. from CETAS) was found to be 99% identical to a sequence of *P*. *falciparum* (M19173); and sample 156 (*Sapajus* sp. from CETAS) was found to be 100% identical to sequences of *Plasmodium malariae* isolates Oumu (AB489196) and Takaboh (AB489195).

None of the 13 NTP samples that were positive in the qPCR and PCR assays selected for the LAMP assays generated amplified products. However, the positive controls (*P*. *falciparum*, *P*. *vivax* and *P*. *malariae*) and the plasmids containing an 84 bp fragment of the 18S rRNA gene of *P*. *falciparum* exhibited amplification curves and dissociation curves with different peaks.

### Serological test (IFA)

The 151 samples of NTP serum that were evaluated were non-reactive against the antigens of *P*. *falciparum* and *P*. *berghei*.

**Fig 3:**
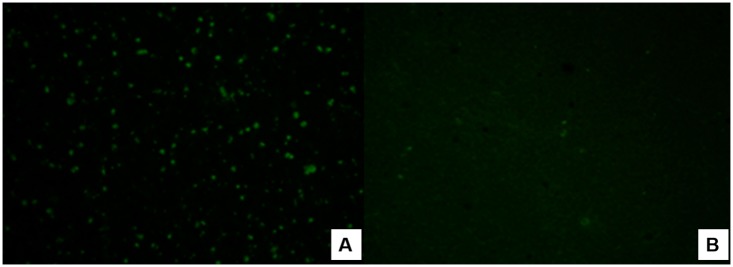
Photomicrographs of the indirect immunofluorescence reaction for IgG antibodies against *P*. *malariae*. A—Positive sample at a dilution of 1:20 from a *Sapajus* specimen (male) at CETAS in São Luís; B—Negative human control serum.

Because of the poor availability of *P*. *malariae* antigen, we selected only ten NTP serum samples that were positive in the PCR and qPCR assays. Among these ten test serum samples from primates of the genus *Sapajus* spp., which were being kept at CETAS in São Luís, four were seropositive for *P*. *malariae* at 1:20 dilutions (Fig 3).

## Discussion

Despite persistent efforts to eradicate malaria, this is far from happening. Although the number of cases has declined worldwide over the past few years, some events put the proposed malaria elimination at risk. Occurrences of asymptomatic infections can reach high rates, such as 68% for *P*. *falciparum* and 12% for *P*. *malariae* [40]. In addition, resistant submicroscopic parasites may make it difficult to eliminate malaria, especially in areas of low transmission [41]. Another challenge is the possibility of transmission of simian malaria to humans, due to the increasing proximity of humans to the wild environment and vice versa. It seems that there is no biological barrier to transmission of some species of *Plasmodium* from NTPs, such as *P*. *brasilianum* and *P*. *simium* [42], and as reported in relation to *P*. *knowlesi* [43]. Studying the prevalence of *Plasmodium* in NTPs in the Americas is a laborious assignment because of the difficulty in catching these animals in the forest environment and collecting biological samples from them, and also because of the complexity of identifying monkey species [44,45].

The present study focused on investigation of occurrences of *Plasmodium* among free-living and captive NTPs in the municipalities of the island of São Luís, state of Maranhão, Brazil. Samples from 161 animals in six different species were analyzed.

Young trophozoites of *Plasmodium* were observed in slides prepared from blood samples from these Neotropical primates, under an light microscope. However, these could not be identified to species taxon because young trophozoite forms are very similar among *Plasmodium* species [27]. Making a diagnosis based on the morphology of *Plasmodium* species infecting nonhuman primates is a challenge and can lead to ambiguous results, even when performed by experienced microscopists. Human blood samples infected with *P*. *knowlesi* were erroneously diagnosed as *P*. *malariae* in Malaysia in 1999 and 2000 [40]. *P*. *malariae* can also be misdiagnosed as *P*. *vivax* from examination of thick blood smears under a microscope, given that the ring shape of the two species is quite similar [46]. The difficulty in differentiating young forms of *Plasmodium* is further increased by low levels of parasitemia, as was found in the present study, in which only one or two trophozoites were identified in each slide. Thus, as occurred in India and Malaysia, many cases of *P*. *malariae* may have been erroneously diagnosed as *P*. *vivax*, in routine thick blood smear microscopy in several countries [43]. In Central and South America, *P*. *brasilianum* and *P*. *simium* infect NTPs, and these have morphological and genetic similarities to *P*. *malariae* and *P*. *vivax*, respectively [11,25,47–49]. In areas of South America in which humans and NTPs coexist, *P*. *malariae* infection cannot be differentiated from *P*. *brasilianum*, or *P*. *vivax* from *P*. *simium*, by means of light microscopy [50]. Even the most widely used molecular diagnostic protocol for diagnosing human malaria [51] is incapable of distinguishing *P*. *malariae* from *P*. *brasilianum*, or *P*. *vivax* from *P*. *simium*. Likewise, *P*. *cynomolgi* is indistinguishable from *P*. *vivax* [52], possibly because the 18S rRNA gene is conserved in these species [49]. In the present study, 47 out of 48 samples were positive for *P*. *malariae/P*. *brasilianum*, according to PCR. One sample showed a band in the genus-specific reaction, but not in the second PCR reaction. However, no sample was positive for *P*. *simium*, although *P*. *vivax* (the equivalent species in humans) is prevalent in the area studied [53–55]. These results confirm that, to date, this species of *Plasmodium* is restricted to Atlantic Forest primates [18,19].

*P*. *malariae/P*. *brasilianum* is the most commonly diagnosed species in primates in the Americas [11,18,19]. However, in humans, it is less prevalent than *P*. *vivax* and *P*. *falciparum* [3]. In the municipalities studied, no human cases of *P*. *malariae* were notified [53–55]. However, this species may be underreported, since it can be misdiagnosed as *P*. *vivax* from examination of thick blood smears [56]. In addition, even sensitive PCR has flaws with regard to diagnosing *P*. *malariae*, because the parasite load is generally close to the detection threshold of the technique. Moreover, other authors have suggested that there are intraspecific variants of the 18S rRNA gene, especially in *P*. *malariae*. Thus, when the target gene is 18S rRNA, failure of PCR to detect *P*. *malariae* is not unlikely, because of probable variations in the sequence of the target region [57,58].

Among selected samples that were known to be positive through qPCR and PCR, none of the DNA samples extracted from NTP blood were positive for LAMP. This was probably because most protocols for LAMP have been standardized using clinical samples from human patients with malaria symptoms [59]. Thus, these would not be capable of detect the low levels of *Plasmodium* parasitemia in field samples from NTPs. The results showed that, at least for *Plasmodium* in the samples from NTPs in the present study, the LAMP technique was insufficiently sensitive.

Trouble in sequencing *Plasmodium* DNA samples in blood samples from NTPs is quite common, since the fragments give rise to low-intensity bands. A few Brazilian studies in the literature have demonstrated successful sequencing, generally through using fragments generated from the genes that code the circumsporozoite protein (CSP) or merozoite surface protein (MSP) [45,60]. More recently, other targets have also been used for sequencing malaria parasites from NTPs, such as using Duffy binding protein [61] and 18S rRNA [20]. However, more commonly, studies have not included sequencing, even when analyzing large numbers of samples [19,62].

Regarding detection of anti-*Plasmodium* antibodies in the present study, four serum samples (n = 151) from *Sapajus* sp. (CETAS, São Luís) that were positive in the qPCR and PCR assays, were reactive against *P*. *malariae* antigens. Therefore, the difficulty in detecting anti-*Plasmodium* antibodies in NTPs is evident, since reactivity in the indirect immunofluorescence antibody test was only observed in the present study at 1:20 dilutions against the *P*. *malariae* antigen. This confirms the results from a study by Duarte et al. [63], on primates in the Cerrado and Atlantic Forest regions (n = 44). These authors did not find that any serum samples from *Callithrix* (n = 44) or *Cebus apella* (n = 56) were reactive to any of the three antigens used (*P*. *falciparum*, *P*. *malariae and P*. *vivax*) using this test. According to these authors, the primate species sampled did not play any important epidemiological role in malaria transmission in the areas that they studied. Attempts to use *P*. *falciparum* and *P*. *berghei* antigens are justified, since cross-reactivity is expected, given that it is known that *Plasmodium* antigens produced in laboratories (especially from animals) are usually used in making the serodiagnosis of human malaria [64].

It is important to note that because CETAS receives animals that were seized throughout the state of Maranhão, which had often been newly trapped from the wild or had been in captivity for more than two years, positivity for *Plasmodium* was high in relation to the animals caught at the Sítio Aguahy (free-living). Although CETAS is located in a populous neighborhood, reforestation of the environment has been taking place over the last five years. Also, the Paciência river passes near to CETAS (80 meters away). This stretch of the river is polluted and has a weak current, and thus it cannot serve as a breeding ground for anophelines. However, the foliage of the adjacent ciliary forest can serve as a breeding ground, and larvae of *Aedes aegypti* have already been found there (result not shown).

Considering the results from this study and the data in the literature on the circulation of malaria in NTPs and in the human population in rural and forest areas [22,23], simian malaria cannot be further neglected with the excuse that it is considered to have low pathogenicity and low prevalence. With lateral transmission between humans and NTPs, of *P*. *malariae/P*. *brasilianum* and *P*. *vivax/P*. *simium* [21,22], eradication of malaria in the Americas is becoming more challenging. Thus, malaria as a zoonotic disease should be seen not only as a public health problem, but also as one of environmental conservation, encompassing preservation of the wild environment, hosts and vectors, in order to preserve the balance in these areas. Through extinction or decreased populations of nonhuman primates, vector mosquitoes have the alternative of replace their primatophilic food source with an anthropophilic source, as has been reported with other hosts [65,66], and thus the disease may come to present epidemiological characteristics of greater severity.

*P*. *brasilianum* and *P*. *simium* can infect human beings [21,22]. Because of continuous and increasing invasion of the wild environment by human populations, the potential for zoonoses should always be considered, as well as the behavior and adaptability of parasites and vectors. Strategies based on further research should be developed for prevention and diagnosis of zoonotic malaria.

## Conclusions

In this study, it was confirmed that *P*. *malariae*/*P*. *brasilianum* circulates in Neotropical primates, with low levels of parasitemia and low levels of antibodies. The importance of these animals as reservoirs of human malaria in the region studied is still unknown. This scenario has an impact on control and elimination of malaria in this region.
